# Multilocus Genotyping of *Giardia duodenalis* in Alpine Musk Deer (*Moschus chrysogaster*) in China

**DOI:** 10.3389/fcimb.2022.856429

**Published:** 2022-04-20

**Authors:** Zhaohui Cui, Qilin Wang, Xiyao Huang, Jiayi Bai, Bingyang Zhu, Bingchen Wang, Xiaohang Guo, Meng Qi, Junqiang Li

**Affiliations:** ^1^Key Laboratory of Biomarker Based Rapid-Detection Technology for Food Safety of Henan Province, Food and Pharmacy College, Xuchang University, Xuchang, China; ^2^College of Animal Science and Technology, Tarim University, Alar, China; ^3^College of Veterinary Medicine, Henan Agricultural University, Zhengzhou, China

**Keywords:** *Giardia duodenalis*, alpine musk deer, multilocus genotyping, zoonotic potential, China

## Abstract

*Giardia duodenalis* is the underlying cause of a significant number of outbreaks of gastrointestinal illness in humans and animals worldwide. The purpose of this study was to elucidate the prevalence and genetic diversity of *G. duodenalis* in captive alpine musk deer (*Moschus chrysogaster*) in China. A total of 202 fecal samples were collected from three farms in Gansu Province, China. Identification of *G. duodenalis* was conducted by nested PCR targeting the genes coding for SSU rRNA, β-giardin (*bg*), glutamate dehydrogenase (*gdh*) and triosephosphate isomerase (*tpi*). The overall prevalence of *G. duodenalis* in captive alpine musk deer in surveyed area was 19.3% (39/202). Two *G. duodenalis* genetic assemblages were identified, namely assemblage A and E. Mixed genotype infections (A+E) were found in 15.4% (6/39) of positive samples. Multilocus genotyping (MLG) analysis of *G. duodenalis* isolates revealed six novel assemblage A MLGs formed by two newly-described MLG-subtypes which belonged to sub-assemblage AI. To the best of our knowledge, this is the first report on MLG of *G. duodenalis* isolates in captive alpine musk deer in China. The presence of zoonotic assemblages and sub-assemblages of *G. duodenalis* in deer species suggests that these animals may potentially act as a reservoir of this protozoan for humans.

## Introduction

*Giardia duodenalis* (also known as *Giardia lamblia* and *Giardia intestinalis*) is the most prevalent protozoan pathogen, commonly found in the intestinal tract of humans and animals worldwide (Adam, 2021). Transmission of *G. duodenalis* infection occurs by several routes either directly (i.e., person-to-person, animal-to-animal, or zoonotic infection) or indirectly (i.e., water or food) (Dixon, 2021). Approximately 280 million people are considered to be infected with *G. duodenalis* worldwide, with infection rates at the range of 8.0–30.0% in developing countries and 0.4–7.5% in developed countries (Feng and Xiao, 2011; Ryan and Zahedi, 2019). Giardiasis is generally a self-limiting clinical illness in humans, whereas it can be threatening to infants, young children, the elderly, institutionalized individuals, travelers, and immunocompromised individuals (Cacciò et al., 2018; Cai et al., 2021). Nitroimidazoles (e.g., metronidazole and tinidazole) are the most commonly drugs used to treat giardiasis, although requiring multiple doses and being often associated with adverse effects (Argüello-García et al., 2020).

To date, according to the reservoir and genetic characteristics of the protozoan, eight *Giardia* species have been recognized (Ryan et al., 2019). However, most studies for both public and veterinary health have focused on the taxonomy, population genetics, and epidemiology of *G. duodenalis* (Li et al., 2017a; Xiao and Feng, 2017). Based on studies employing iso-enzymatic and nucleic acid polymorphisms, *G. duodenalis* is known as a multispecies complex, consisting of eight genetic assemblages (A–H) considering different host distribution (Cacciò et al., 2018); assemblages A and B are commonly found in humans and occasionally in other mammals; assemblages C and D are often found in canids; assemblage E mainly infects ungulates; assemblages F, G, and H are specific to cats, rodents and pinnipeds, respectively (Ryan and Cacciò, 2013; Cai et al., 2021). Furthermore, putative sub-assemblages have been identified within assemblage A (AI–III) and assemblage B (BIII and BIV) using a multilocus genotyping (MLG) approach (Capewell et al., 2021).

China has the largest wild and captive populations of alpine musk deer (*Moschus chrysogaster*) in the world, which are mainly distributed in the Qinghai Tibet Plateau, Sichuan and Gansu Province (Jiang et al., 2021). Musk, produced by adult male alpine musk deer, is an important raw material for preparations of the traditional Chinese medicine and the fragrance industry. However, illegal hunting, habitat fragmentation, and other human activities have decimated wild alpine musk deer populations in China (Cai et al., 2020). For these reasons, the alpine musk deer has been listed as an endangered species by the International Union for Conservation of Nature (IUCN) and as category I-protected wild animal in China. Although the Chinese government has encouraged enterprises to participate in programs of breeding of captive alpine musk deer, gastrointestinal infections are the most significant threats to population growth and breeding scale whose fatality rate is approximately 30% (Li et al., 2017b). Currently, little information is available on the prevalence and genetic characteristics of *G. duodenalis* in cervids in China.

Thus, the aim of the present study was to investigate the prevalence and genetic diversity of *G. duodenalis* in captive alpine musk deer. The findings discussed herein provide insights into the development of preventive measures against *Giardia* infection.

## Material and Methods

### Ethics Statement

This study was performed with strict adherence to the recommendations of the Guide for the Care and Use of Laboratory Animals of the Ministry of Health, China. The research protocol was reviewed and approved by the Research Ethics Committee of Tarim University (approval no. ECTU 2020-0013). Farm owners’ consent was obtained prior to proceeding to fecal sample collection from selected animals.

### Samples

**Figure 1:**
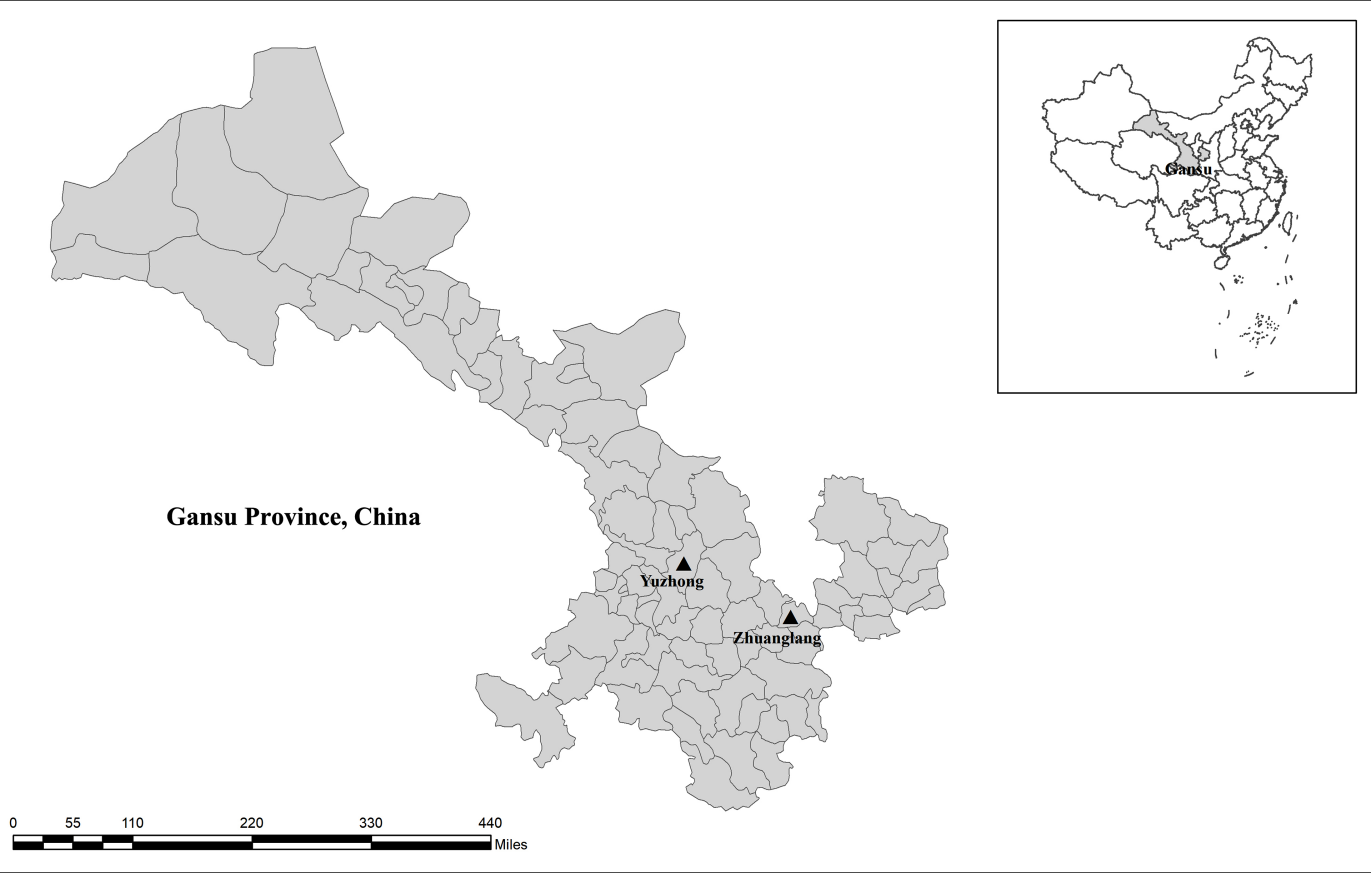
Sampling sites. No copyright permission was required. The figure was designed with the software ArcGIS 10.2. The map has been originally modified and assembled according to permission and attribution guidelines of the National Geomatics Center of China (http://www.ngcc.cn).

In September 2020, 202 fecal samples were collected from three farms in Gansu Province, China (**Figure 1**). Alpine musk deer animals were shed-fed and housed in separate breeding houses according to age. Fresh fecal samples were taken from the soil immediately after defecation using a sterile disposal latex glove, ensure absence of exogenous contamination. Subsequently, samples were placed individually into a disposable plastic container, recorded the date, site, age, and number. All animals from which fecal samples were obtained were apparently in good health with no signs of diarrhea at the time of sample collection. Samples were transferred to the laboratory in an insulated container on ice and stored at 4°C prior to DNA extraction.

### DNA Extraction and Genotyping

Genomic DNA was extracted from approximately 200 mg of each precipitated sample using the E.Z.N.A.^®^ Stool DNA kit (Omega Bio-tek Inc., Norcross, GA, USA), according to manufacturer’s instructions. The extracted DNA was stored at -20°C until PCR assay. Four genes were used for *G. duodenalis* genotyping by nested PCR, namely SSU rRNA, β-giardin (*bg*), glutamate dehydrogenase (*gdh*), and triosephosphate isomerase (*tpi*) (**Table 1**). Positive (DNA from an isolate known to harbor the four surveyed loci) and negative (reagent-grade water) controls were included in each PCR amplification.

**Table 1:** Primer sequences and reaction conditions used in nested PCR amplifications.

Target Gene	Primer sequences (5’- 3’)	Annealing	Target size	Reference
	Gia2029 (AAGTGTGGTGCAGACGGACTC)	55°C		
SSU rRNA	Gia2150c (CTGCTGCCGTCCTTGGATGT)			
	RH11 (CATCCGGTCGATCCTGCC)	59°C	292 bp	(Appelbee et al., 2003)
	RH4 (AGTCGAACCCTGATTCTCCGCCCAGG)			
	AL3543 (AAATIATGCCTGCTCGTCG)	50°C		
*tpi*	AL3546 (CAAACCTTITCCGCAAACC)			
	AL3544 (CCCTTCATCGGIGGTAACTT)	50°C	530 bp	(Sulaiman et al., 2003)
	AL3545 (GTGGCCACCACICCCGTGCC)			
	GDH1 (TTCCGTRTYCAGTACAACTC)	50°C		
*gdh*	GDH2 (ACCTCGTTCTGRGTGGCGCA)			
	GDH3 (ATGACYGAGCTYCAGAGGCACGT)	50°C	530 bp	(Cacciò et al., 2008)
	GDH4 (GTGGCGCARGGCATGATGCA)			
	G7 (AAGCCCGACGACCTCACCCGCAGTGC)	58°C		
*bg*	G759 (GAGGCCGCCCTGGATCTTCGAGACGAC)			
	2005F (GAACGAACGAGATCGAGGTCCG)	55°C	511 bp	(Lalle et al., 2005)
	2005R (CTCGACGAGCTTCGTGTT)			

### Sequence and Phylogenetic Analysis

All positive secondary PCR products from SSU rRNA, *bg*, *gdh*, and *tpi* genes were bi-sequenced by GENEWIZ (Suzhou, China). Nucleotide sequences were aligned and edited with DNAstar Lasergene Editseq 7.1.0 (https://www.dnastar.com/software/lasergene/) and Chromas Pro 2.1.10 (http://technelysium.com.au/wp/chromaspro/). Genotypes and subtypes of *G. duodenalis* were determined by aligning reference sequences available in NCBI GenBank database using ClustalX 2.1 (http://www.clustal.org/). To determine genetic diversity among the isolates, concatenated sequences (*bg*-*tpi*-*gdh*) from each isolate at the three analyzed loci were aligned with reference sequences. Neighbor-joining (NJ) analysis was performed using MEGA 7.0 (http://www.megasoftware.net/) to infer the phylogenetic relationships of concatenated sequences based on the Kimura-2 parameter model.

### Nucleotide Sequence Accession Numbers

Representative nucleotide sequences of *bg* and *tpi* genes of *G. duodenalis* are available in the NCBI GenBank database under the accession numbers OM273018-OM273020, respectively.

## Results

### Prevalence and Assemblages of *G. duodenalis*

Overall, a total of 39 samples (19.3%, 39/202) were confirmed to be *G. duodenalis* by PCR at the SSU rRNA locus (**Table 2**). Prevalence rate by region was as follows: Yuzhong A (22.2%, 8/36), Yuzhong B (24.6%, 16/65), and Zhuanglang (14.9%, 15/101). In addition, the infection was numerically more frequent in adults (>1 year, 20.6%, 35/170) compared to young animals (<6 month, 12.5%, 4/32). Subsequently, all *G. duodenalis*-positive samples were genotyped by MLG of SSU rRNA, *bg*, *tpi* and *gdh* genes. Two *G. duodenalis* genetic assemblages were identified among samples: assemblage A (72.2%, 26/39) and assemblage E (17.9%, 7/39). Mixed genotype infections (A+E) were found in 6 of 39 samples.

**Table 2:** Prevalence of *G. duodenalis* by location in Gansu Province, China.

Farm	N/T (%)	assemblages (n)	SSU rRNA (n)	*tpi* (n)	*gdh* (n)	*bg* (n)
Yuzhong A	8/36 (22.2)	A (7), E (1)	A (7), E (1)	A (3)	A (2)	A (2)
Yuzhong B	16/65 (24.6)	A (9), E (2), A+E (5)	A (11), E (5)	A (11)	A (1), E (2)	A (4), E (2)
Zhuanglang	15/101 (14.9)	A (10), E (4), A+E (1)	A (10), E (5)	A (9), E (1)	A (4), E (1)	A (7), E (3)
Total	39/202 (19.3)	A (26), E (7), A+E (6)	A (28), E (11)	A (23), E (1)	A (7), E (3)	A (13), E (5)
**Age**						
<6 month	4/32 (12.5)	A (1), E (2), A+E (1)	A (1), E (3)	A (1)	E (1)	E (2)
>1 year	35/170 (20.6)	A (25), E (5), A+E (5)	A (27), E (8)	A (22), E (1)	A (7), E (2)	A (13), E (3)

N, number of positives for G. duodenalis; T, total analysis samples.

### Polymorphisms at Single Loci

Amplification of the *bg* gene was obtained from 18 of 39 *G. duodenalis*-positive isolates; among these, 13/18 (72.2%) of isolates were identified as belonging to genetic assemblage A and 5/18 (27.8%) of assemblage E (**Table 3**). Within assemblage A isolates, three subtypes were formed and designated as A1 (n=4), A2 (n=1), and A3 (n=8). Compared to the sequence MK610391, A1 sequences exhibited one single-nucleotide polymorphism (SNP) (C327T), whereas A2 sequence contained three SNPs (T302C, G308A, and C327T). A3 sequences were identical to MK610392. Moreover, the five assemblage E sequences were identical to the sequence MK610387.

**Table 3:** Multi-locus characterization of *G. duodenalis* isolates in alpine musk deer in China based on *bg*, *gdh* and *tpi* genes.

Isolate Code	*bg*	*tpi*	*gdh*	MLG Type
22	PN	A1	A1	
32	A1 (OM273018)	A1	PN	
47,80	A1	A1	A1	AI-novel 1
65	A1	A1	PN	
71	PN	A1	PN	
78	PN	A1	PN	
79	E	A1	PN	
81	PN	A1	PN	
86	E	A2 (OM273020)	E	Excluded
100	PN	A1	E	
104	PN	A1	PN	
115	A2 (OM273019)	A1	PN	
120	A3	A3	PN	
147	E	A2	PN	
152	E	PN	E	
157,172,195,207	A3	A3	A2	AI-novel 2
163	PN	E1	PN	
173	E	PN	PN	
182	A3	A3	PN	
199	PN	A3	PN	
204	A3	A3	PN	
217	A3	A3	PN	

PN, PCR negative.

Sequence analysis of the *tpi* locus revealed that 23 out of 24 successfully amplified isolates were identified as assemblage A, whereas only one was classified as assemblage E. The single assemblage E sequence was 100% identical to the sequence KT922262. Among assemblage A sequences, A1 (n=12) and A3 (n=9) sequences were identical to the sequences MK639171 and MK639172, respectively. In addition, A2 (n=2) sequences showed a SNP (C21T) compared to the sequence MK639173. At the *gdh* locus, seven and three isolates were successfully amplified and identified as assemblage A and E, respectively. The subtypes A1 (n=3) and A2 (n=4) were consistent with the sequences MN047217 and the MK645799, respectively. Moreover, the three assemblage E sequences were identical to the sequence MK645786.

### MLG and Phylogenetic Analysis

**Figure 2:**
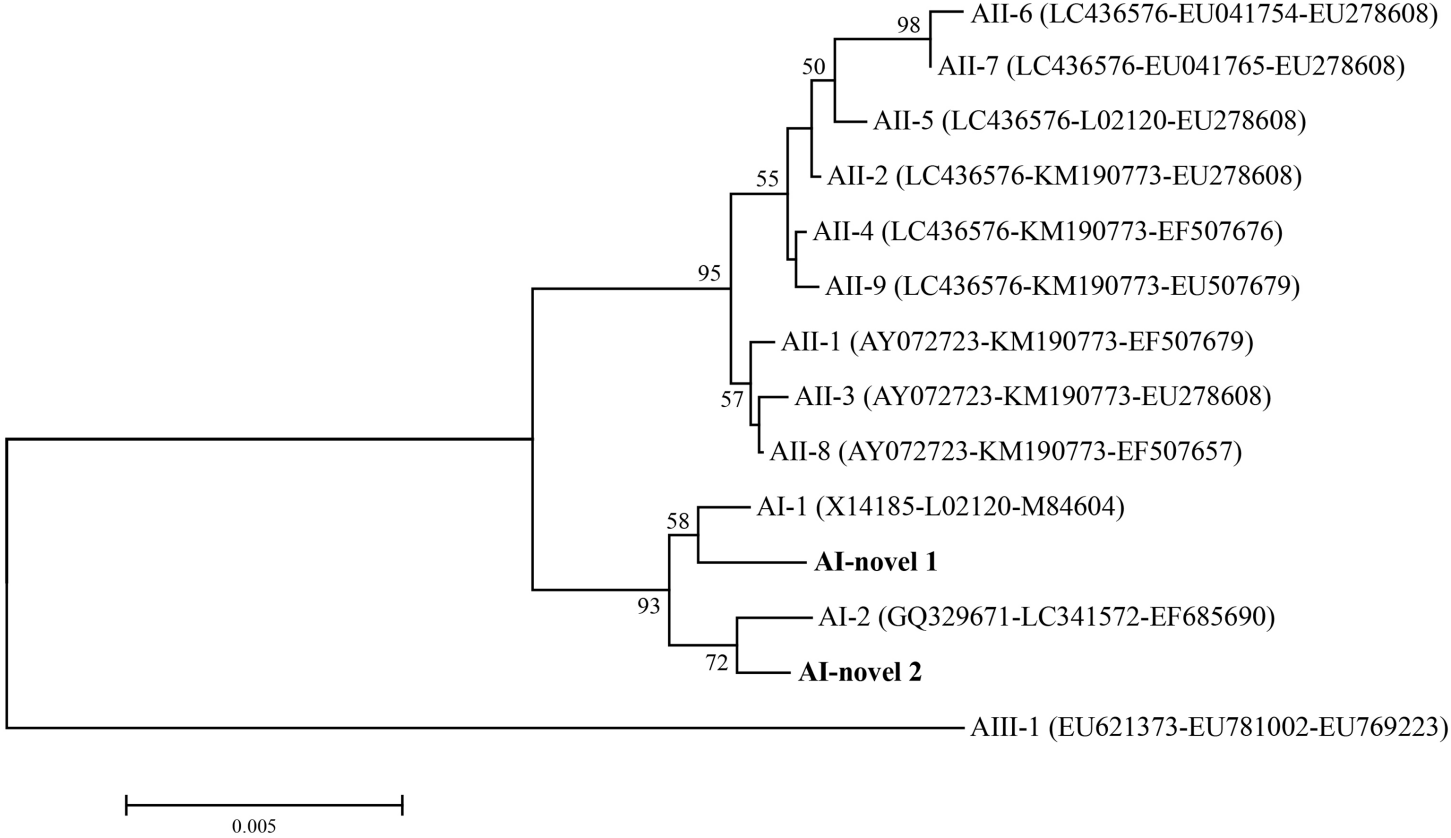
Phylogenetic relationships among *G. duodenalis* assemblage A isolates inferred by neighbor-joining analysis based on concatenated datasets for *bg*, *tpi* and *gdh* nucleotide sequences. Bootstrap values greater than 50% from 1000 replicates were shown on nodes. The novel MLGs in this study were indicated in bold.

In total, seven isolates were successfully sequenced at *bg*, *tpi* and *gdh* loci, and formed six assemblage A MLGs after removal of sequences of mixed infection samples (**Table 3**). The six identified assemblage A MLGs were composed of two novel MLG-subtypes (AI-novel 1 and AI-novel 2); AI-novel 1 was found in two isolates, whereas AI-novel 2 was identified in four isolates. Phylogenetic relationships of assemblage A MLGs with reference genotypes are shown in **Figure 2**. Based on phylogenetic analysis, all assemblage A MLGs identified herein were clustered in the MLG AI branch, with MLG AI-novel 1 clustered closer to MLG AI-1, whereas MLG AI-novel 2 clustered closer to MLG AI-2.

## Discussion

Six species of musk deer (*Moschus* spp.) have historically been bred in China, which include siberian musk deer (*M. moschiferus*), forest musk deer (*M. berezovskii*), black musk deer (*M. fuscus*), alpine musk deer (*M. chrysogaster*), himalayan musk deer (*M. leucogaster*), and anhui musk deer (*M. anhuiensis*) (Fan et al., 2018). In previous studies, deer have been considered as a major reservoir of viruses, bacteria, and parasites for humans and livestock (Böhm et al., 2007; Mehrpad et al., 2018). In particular, a recent study has suggested the potential emergence of a new reservoir of SARS-CoV-2 viruses in free-ranging white-tailed deer, which may open new pathways for evolution, transmission to other wildlife species, and potential spillback of novel variants to humans (Hale et al., 2022).

*Giardia* spp. infects a broad range of hosts including humans, livestock, companion animals, wildlife and birds (Ryan et al., 2021). However, information on the distribution, molecular characteristics and zoonotic potential of *Giardia* in cervids is scant. To date, *G. duodenalis* infections in cervids have been reported in several countries, including Australia, Bangladesh, Canada, Croatia, Italy, Japan, Netherlands, Norway, Poland, Spain, Sweden, USA and China, with the infection rates ranging from 0.6% to 24.0% (**Table 4**). Interestingly, the rodent-specific species *Giardia microti* has been isolated in roe deer (*Capreolus capreolus*) in Croatia (Beck et al., 2011). In the present study, the prevalence of *G. duodenalis* was 19.3% (39/202) in alpine musk deer, which is higher than that reported in sika deer (0.6% and 0.8%) and forest musk deer (2.2%) in China. The discrepancies in infection rates of *G. duodenalis* may be related to the differences in geographical location, sampling season, animal species, and sample size. To the best of our knowledge, this is the first study to isolate and characterize *G. duodenalis* from alpine musk deer in China using MLG.

**Table 4:** *Giardia duodenalis* infection rates and genotypes in cervids worldwide.

Location	Host	Positive % (N/T)	Assemblage (n)	Sub-Assemblage (n)	Reference
Australia	Sambar deer, red deer, fallow deer	0.6 (10/1563)	A (10)	A-I (1), A-III (9)	(Koehler et al., 2016)
Bangladesh	Spotted deer	3.3 (1/30)	A (1)		(Karim et al., 2021)
Canada	Boreal caribou	2.0 (3/149)			(Johnson et al., 2010)
China	Sika deer	0.8 (5/662)	E (5)		(Huang et al., 2018)
	Forest musk deer	2.2 (5/223)	A (2), E (3)		(Song et al., 2018)
	Sika deer	0.6 (5/818)	A (2), E (3)		(Ma et al., 2021)
	Alpine musk deer	19.3 (39/202)	A (22), E (5), A+E (6)		**This study**
Croatia	Red deer	1.1 (4/374)	A (3), D (1)		(Beck et al., 2011)
	Roe deer	24.0 (5/21)	A (2), D (2), *G. microti* (1)		(Beck et al., 2011)
Italy	Fallow deer	11.5 (16/139)	A (8)	A-I (8)	(Lalle et al., 2007)
	Fallow deer		A (8)	A-III (8)	(Cacciò et al., 2008)
Japan	Sika deer	0.7% (2/271)	A (2)		(Yamazaki et al., 2018)
Netherlands	Roe deer		A (1)		(van der Giessen et al., 2006)
Norway	Reindeer	5.0% (6/114)	A (6)	AI (6)	(Idland et al., 2021)
	Reindeer		A (6)		(Robertson et al., 2007)
	Moose		A (13)		(Robertson et al., 2007)
	Red deer	1.7 (5/289)			(Hamnes et al., 2006)
	Roe deer	15.5 (45/291)			(Hamnes et al., 2006)
	Reindeer	7.1 (11/115)			(Hamnes et al., 2006)
	Moose	12.3 (56/455)			(Hamnes et al., 2006)
Poland	Red deer	1.6 (1/61)	A (1)	A-III (1)	(Solarczyk et al., 2012)
	Roe deer	4.0% (2/50)	A (2)	A-I (2)	(Solarczyk et al., 2012)
	Red deer	17.9 (5/28)	B (4)		(Stojecki et al., 2015)
	Roe deer	22.9 (11/48)	B (8)		(Stojecki et al., 2015)
	Moose	17.0 (4/23)			(Stojecki et al., 2015)
Spain	Roe deer	8.9 (19/212)	A (7)	A-II (7)	(García-Presedo et al., 2013)
	Roe deer	5.4 (12/224)			(Castro-Hermida et al., 2011b)
	Deer	7.7 (14/181)			(Castro-Hermida et al., 2011a)
	Fallow deer		A (1), E (1)		(Lebbad et al., 2010)
	Moose		A (1)		(Lebbad et al., 2010)
USA	White-tailed deer	1.3 (1/80)	A (1)		(Santin and Fayer, 2015)
	White-tailed deer	1.3 (5/394)			(Rickard et al., 1999)
	White-tailed deer	3.8 (1/26)	A (1)		(Trout et al., 2003)
	Reindeer		A (1)		(Miska et al., 2009)

N=number of positives for G. duodenalis; T, total analysis samples.

Methods based on sequence analysis of SSU rRNA, *gdh*, *bg* and *tpi* genes have currently been widely used for genotyping *G. duodenalis* isolates from human and animal samples in order to obtain high-sequencing resolution (Feng and Xiao, 2011). To date, molecular studies have identified *G. duodenalis* in fallow deer, forest musk deer, moose, red deer, reindeer, roe deer, sambar deer, spotted deer and white-tailed deer with a worldwide distribution (**Table 4**). In addition to zoonotic assemblages A and B, other *G. duodenalis* assemblages including E (mainly found in hoofed mammals) and D (mainly found in canines) have also been reported occasionally in these animal hosts (Beck et al., 2011; Huang et al., 2018), which suggests potential transmission routs of *G. duodenalis* assemblages between humans, livestock, companion animals and cervids. In the present study, both assemblage A and E were identified, and assemblage A was the predominant genotype. Mixed infections were observed in alpine musk deer at both assemblage and sub-assemblage levels, which may be a result of infection with *Giardia* parasites with different genetic profiles. Assemblages A is responsible for most giardiasis cases in humans, especially in South America and the Middle East (Xiao and Feng, 2017; Ryan et al., 2021). Interestingly, the host-adapted genotype assemblage E which was approximately 87% similar to assemblages A in genome, has been reported in at least 57 human giardiasis cases in Brazil, Egypt, Vietnam, Australia and New Zealand (Abdel-Moein and Saeed, 2016; Fantinatti et al., 2016; Zahedi et al., 2017; Garcia-R et al., 2021; Iwashita et al., 2021). Collectively, the presence of zoonotic assemblages of *G. duodenalis* in alpine musk deer in China suggests that these animals may potentially act as a reservoir of *G. duodenalis* for humans.

Moreover, in order to elucidate the genetic diversity of *G. duodenalis* in alpine musk deer, positive samples identified in the present were subjected for sub-genotyping by MLG. Moderate genetic variation was observed within assemblage A sequences, whereas no genetic variation was noticed within assemblage E sequences, which may be due to the low allelic sequence heterozygosity (ASH) in the genomes of assemblages A and E (Kooyman et al., 2019). In addition, the six assemblage A MLGs were composed of two novel MLG-subtypes which belonged to sub-assemblage AI. Among the three sub-assemblages within assemblage A, sub-assemblage AI is most commonly found in animals, whereas sub-assemblage AII is mostly found in humans; sub-assemblage AIII is rare and has been found in wild ruminants and two human giardiasis cases in Romania and New Zealand (Feng and Xiao, 2011; Cai et al., 2021; Ryan et al., 2021). In published studies, both sub-assemblage AI, AII and AIII were identified in various deer (**Table 4**). Further studies based on MLG analysis are necessary to gain a better understanding on the potential role of deer in the zoonotic transmission of *G. duodenalis*.

## Conclusions

In conclusion, this is the first report of *G. duodenalis* in alpine musk deer with a high prevalence in China. Two *G. duodenalis* assemblages were identified, assemblage A and E. Moderate genetic diversity was observed within assemblage A sequences based on MLG analysis. Six assemblage A MLGs were identified which were composed of two novel MLG-subtypes belonging to sub-assemblage AI. Collectively, zoonotic assemblages of *G. duodenalis* identified in the present study point out that alpine musk deer may potentially act as reservoirs of this protozoan to humans.

## Data Availability Statement

The datasets presented in this study can be found in online repositories. The names of the repository/repositories and accession number(s) can be found in the article/supplementary material.

## Ethics Statement

The animal study was reviewed and approved by Research Ethics Committee of Tarim University. Written informed consent was obtained from the owners for the participation of their animals in this study.

## Author Contributions

ZC, QW, XH, JB, BZ, BW, and XG performed the experiments. ZC drafted the manuscript. MQ and JL critically revised the manuscript. All authors contributed to the article and approved the submitted version.

## Funding

The research was funded by the National Natural Science Foundation of China (32102689), Science and Technology Planning Project of Henan Province (222102110240), the Program for Young and Middle-aged Leading Science, Technology, and Innovation of Xinjiang Production & Construction Group (2018CB034), and Scientific research project of Xuchang University (2022GJPY009 and 2022YB034).

## Conflict of Interest

The authors declare that the research was conducted in the absence of any commercial or financial relationships that could be construed as a potential conflict of interest.

## Publisher’s Note

All claims expressed in this article are solely those of the authors and do not necessarily represent those of their affiliated organizations, or those of the publisher, the editors and the reviewers. Any product that may be evaluated in this article, or claim that may be made by its manufacturer, is not guaranteed or endorsed by the publisher.
